# Comparative Study of Transcriptome Profiles of Mouse Livers and Skins Infected by Fork-Tailed or Non-Fork-Tailed *Schistosoma japonicum*

**DOI:** 10.3389/fmicb.2017.01648

**Published:** 2017-08-30

**Authors:** Yan Yang, Jun-Jun He, Shuang Hu, Hua Chang, Xun Xiang, Jian-Fa Yang, Feng-Cai Zou

**Affiliations:** ^1^Key Laboratory of Veterinary Public Health of Yunnan Province, College of Veterinary Medicine, Yunnan Agricultural University Kunming, China; ^2^State Key Laboratory of Veterinary Etiological Biology, Key Laboratory of Veterinary Parasitology of Gansu Province, Lanzhou Veterinary Research Institute, Chinese Academy of Agricultural Sciences Lanzhou, China

**Keywords:** *Schistosoma japonicum*, transcriptome profiles, liver, skin, morphology distinct cercariae

## Abstract

*Schistosoma japonicum* (*S. japonicum*) is a worldwide spread pathogen which penetrates host skin and then induces several diseases in infected host, such as fibrosis, formation of granulomas, hepatocirrhosis, and hepatomegaly. In present study, for the first time, transcriptomic profiles of mouse livers and skins infected by fork-tailed *S. japonicum* cercaria or non-fork-tailed *S. japonicum* cercaria were analyzed by using RNA-seq. The present findings demonstrated that transcriptomic landscapes of livers and skins infected by fork-tailed *S. japonicum* cercaria or non-fork-tailed *S. japonicum* cercaria were different. *S. japonicum* has great influence on hepatic metabolic processes. Fork-tailed *S. japonicum* cercaria upregulated hepatic metabolic processes, while non-fork-tailed *S. japonicum* cercaria downregulated hepatic metabolic processes. For the metabolism process or the metabolism enzyme expressional change, the pharmacokinetics of host could be changed during *S. japonicum* infection, regardless the biotypes of *S. japonicum* cercariae. The changes of infected skins focused on upregulation of immune response. During the *S. japonicum* skin infection period, fork-tailed *S. japonicum* cercaria infection induced stronger immune response comparing with that immune response triggered by non-fork-tailed *S. japonicum* cercaria. The transcription factor enrichment analysis showed that Irf7, Stat1 and Stat2 could play important roles in gene regulation during fork-tailed *S. japonicum* cercaria infection.

## Introduction

*Schistosoma japonicum* (*S. japonicum*) is a worldwide spread blood fluke which prevalent among dozens of countries, including Cambodia, Brazil, Syria, Turkey, Japan, and China. The parasite has a very wide definitive host range, including human, ruminant, carnivore and a lot of domestic animals, causing serious harms to human being and animals. Over 200 million people were suffered from schistosomiasis annually (Chitsulo et al., 2000). The infected hosts could show several clinical symptoms, such as infertility, abortion, stomachache, diarrhea, fibrosis, formation of granulomas, hepatocirrhosis, hepatomegaly, and splenomegaly.

*S. japonicum* lives a complex life cycle which consist of several developmental stages, including adult worm, egg, daughter sporocyst, miracidia, cercaria, schistosomula (Gobert et al., 2009). The adult worm shed egg to outside world through definitive host defecating. After for several hours hatching, miracidia burst out from the egg shell and develop into cercaria in the intermediate host snail (*Oncomelania*). Mature cercaria escape from *Oncomelania*, infecting definitive host through skin contact and then develop into schistosomula and adult worm. Adult female *S. japonicum* can lay egg in the liver of infected host and then induces granulomas. Several reports have confirmed that *S. japonicum* infection can alter host gene expression and then jeopardize host. For example, IL-4 (Oswald et al., 2001) and IL-13 (Bartley et al., 2006) were reported to be upregulated during *S. japonicum* infection. The alterations of these cytokines are connected with host pathological change closely. For example, inhibiting the expression of IL-4 greatly reduces fibrosis in infected host (Cheever et al., 1995), while upregulation of IL-13 plays an important role in the formation of granuloma-associated fibrosis (Chiaramonte et al., 2003; Mentink-Kane et al., 2004). In these studies, the objects were focused on specific cytokines, such as IL-4 and IL-13. With the deepening of research, we realize that the final result of cytokine response is not only depend on cytokine expressional change but also on its receptor expression. For example, during pathogen infection, some cytokines could be upregulated, while their receptors could be downregulated (He et al., 2016). Studying expressions of a few genes that are altered during *S. japonicum* infection will lead to a false result. So it is urgent for us to know about the global gene expressional change during *S. japonicum* infection. Transcriptomic technology shows great advantage in studying global gene expressional change, researching the interactions between hosts and pathogens, revealing the pathogenic mechanism of pathogens. During long time evolution, morphology distinct cercariae have evolved. For example, non-fork-tailed cercaria and fork-tailed cercaria of *S. japonicum* are discovered in Yunnan province, China. According to unpublished data, non-fork-tailed cercaria and fork-tailed cercaria show different influences on infected host. Although the transcriptomes of *S. japonicum* have been widely researched (Gobert et al., 2009; Wang et al., 2015; Yang et al., 2015; Cai et al., 2016) and the transcriptomic changes of infected tissues or host cells have been reported (Chuah et al., 2013; Cai et al., 2017; Chen et al., 2017b; Luo et al., 2017), the comparative transcriptomic analysis between morphology distinct cercaria infecting tissues has not been researched. In order to discover the different features between fork-tailed cercaria infecting tissues and non-fork-tailed cercaria infecting tissues, revealing how the infected tissues response to morphology distinct cercariae, comparative analysis of host transcriptomic profiles during fork-tailed *S. japonicum* cercaria and non-fork-tailed *S. japonicum* cercaria was performed.

## Materials and Methods

### Ethics Statement

All animals were handled strictly according to the Animal Ethics Procedures and Guidelines of the People’s Republic of China. The study was reviewed and approved by the Animal Ethics Committee of Yunnan Agricultural University.

### *Schistosoma japonicum* Infection and Sample Collection

Fork-tailed *S. japonicum* cercaria and non-fork-tailed *S. japonicum* cercaria were isolated and maintained by our laboratory. In present study, 12 SPF kunming mice were assigned to six groups, including non-fork-tailed *S. japonicum* cercaria infecting skin (S-nonFT), fork-tailed *S. japonicum* cercaria infecting skin (S-FT), non-fork-tailed *S. japonicum* cercaria infecting liver (L-nonFT), fork-tailed *S. japonicum* cercaria infecting liver (L-FT), non-infected skin control group (SC) and non-infected liver control group (LC). Two replicates were assigned to each group. In the infected groups, 100 cercariae were used for subcutaneous infection. 36 h post infection, mice skins (including infected groups S-nonFT, S-FT and control group SC) were individually harvested post-euthanasia for transcriptomic analysis. Ten days post infection, mice livers (including infected groups L-nonFT, L-FT, and control group LC) were harvested post-euthanasia for transcriptomic analysis. All collected tissues were cryopreserved at -80°C until used for RNA extractions and transcriptomic library constructions.

### RNA Extraction, Qualification and Transcriptomic Library Construction

Total RNA was prepared individually from the mice skin or liver by using TRIzol Reagent according to the manufacturer’s protocol (Invitrogen Co. Ltd.). The RNA prepare protocol details are described as following: (1) 100 mg of cryopreserved tissue were grinded into powders by using liquid nitrogen; (2) 1 ml TRIzol Reagent was added to lysis of the powers followed by adding of 0.5 mL of isopropanol to the aqueous phase; (3) incubated for 10 min; (4) Centrifuge for 10 min at 12,000 ×*g* at 4°C and then discarded the supernatant; (5) resuspend the pellet in 1 mL of 75% ethanol followed by centrifuge for 5 min at 7500 ×*g* at 4°C; (6) discard the supernatant and air dry the RNA pellet for 10 min; (7) resuspend the pellet in 50 μL of RNase-free water and stored at -80°C until use. The quality and quantity of extracted RNAs were tested by using Agilent 2100 Bioanalyzer (Agilent Technologies, Santa Clara, CA, United States) and Nanodrop 2000 (Thermo Scientific, Wilmington, DE, United States) individually. Before RNA library construction and RNA sequencing, the RNAs that RIN (RNA integrity number) >7.0 were treated with 20 units of RNase-Free DNase (Ambion, Shanghai, China) to remove residual genomic DNA. Illumina TruSeq RNA Sample Preparation Kit (Illumina, San Diego, CA, United States) was used for constructions of transcriptomic libraries according to manufacturer’s instructions. Briefly, (1) the mRNA was isolated by using magnetic beads with Oligo (dT); (2) the mRNA was fragmented and then cDNA was synthesized using the mRNA fragments as templates; (3) sequencing adaptor was added to the cDNA fragment and then used the fragment for agarose gel electrophoresis; (4) After agarose gel electrophoresis, the suitable fragments (200 bp) were selected for PCR amplification; (5) ABI StepOnePlus Real-Time PCR System was used in quantification and qualification of the sample library.

### RNA Sequencing, Reads Mapping, Gene Identification and Bioinformatics Analyses of Differentially Expressed Genes (DEGs)

The constructed transcriptomic libraries were sequenced by using Illumina HiSeq 2000 sequencing machine. The raw sequencing data are available at SRA database of NCBI (access number: PRJNA397519). Q20 (99% sequencing accuracy) was used for raw data filtering which discarded the reads with low sequencing quality. After the raw data filtering, the adapters of reads with high sequencing quality were removed and then the clean reads were mapped to the mouse reference genome (mm10) by using BWA and mapped to reference gene by using Bowtie. The expression level of gene was analyzed with FPKM method. NOIseq R package was used for statistical analysis and identification of differentially expressed gene. Differentially expressed gene was defined as fold change ≥2, probability value calculated by NOIseq R package ≥0.8 (Tarazona et al., 2012; Seok et al., 2013; Tanaka et al., 2013; Yan et al., 2013). Gene Ontology (GO) was applied for gene functional annotation and enrichment. Kyoto Encyclopedia of Genes and Genomes database (KEGG) was applied for pathway annotation and enrichment. Significantly enriched GO term or KEGG pathway was defined using hypergeometric test followed by FDR correction (Benjamini and Hochberg, 1995). The FDR corrected *P*-value < 0.01 was used as cutoff of significantly enrichment. Comparative Toxicogenomics Database^1^ was applied for analysis of xenobiotics or drugs that are metabolized by differentially expressed enzymes (Davis et al., 2017). Transcription factor enrichment of differentially expressed gene was analyzed by Pscan (Zambelli et al., 2009).

### Validation of Gene Expression Results of RNA Sequencing

Real-time quantitative PCR (Q-PCR) was applied for validating results of RNA sequencing. The RNAs that used for RNA-seq were reverse-transcribed to cDNA by using PrimeScriptTM RT reagent Kit (TaKaRa, Dalian, China). Nine genes were randomly selected for Q-PCR validation and Actb was chosen as endogenous reference gene. The primers used for Q-PCR are listed in **Table 1**. SYBR green (TaKaRa, Dalian, China) was used for fluorescence intensity detection. Melting curve analysis was performed to ensure the specificities of Q-PCR reactions. The Q-PCR cycle conditions were as follows: 95°C for 3 min followed by 40 cycles of 95°C for 30 s, 56°C for 30 s, 72°C for 2 min; melting curve analysis ranged from 72 to 95°C to ensure that specific product was amplified in each reaction. The 2^-ΔΔC_T_^ relative expression calculating method was used to calculate the relative gene expression (Livak and Schmittgen, 2001).

**Table 1:** Primers used of quantitative PCR (Q-PCR).

Primer names	Sequence (5′->3′)	Product size
Actb-F	GAGACCTTCAACACCCCAGCC	194 bp
Actb-R	GGCCATCTCTTGCTCGAAGTC	
Ces2c-F	CCAGTATCGTCTGGGTGTCC	122 bp
Ces2c-R	CAAAGTGAGCGATGTTCTGC	
CD74-F	GTCTCTGTCCTGGTGGCTCT	107 bp
CD74-R	AGGTTCTGGGAGGTGATGG	
Cmbl-F	TCAGGTTGAGCACATCAAGG	119 bp
Cmbl-R	CCATTTCTGGCAATCATGTC	
Eci 2-F	GCCTCTGGTTGCGGTAGTAA	113 bp
Eci 2-R	GGCTGAATGGAGTATGAAACG	
Ftl1-F	CATCAAGAAGATGGGCAACC	110 bp
Ftl1-R	GGCGCTCAAAGAGATACTCG	
HBB-BS-F	GTGAACGCCGATGAAGTTG	108 bp
HBB-BS-R	AGCAGAGGCAGAGGATAGGTC	
Irgm1-F	AGTTCAGCAGGTAGCCCAGA	110 bp
Irgm1-R	TCAGCCTCAGTTTCCAGTCC	
Psme1-F	AGCCAAGGTGGATGTGTTCC	172 bp
Psme1-R	TGGATCGGGTACTGGGATGT	
Mup17-F	CTCTATGGCCGAGAACCAGA	113 bp
Mup17-R	CGATTGGCATTGGATAGGTC	

## Results

### RNA Extraction, RNA-seq and Differentially Expressed Gene

**FIGURE 1:**
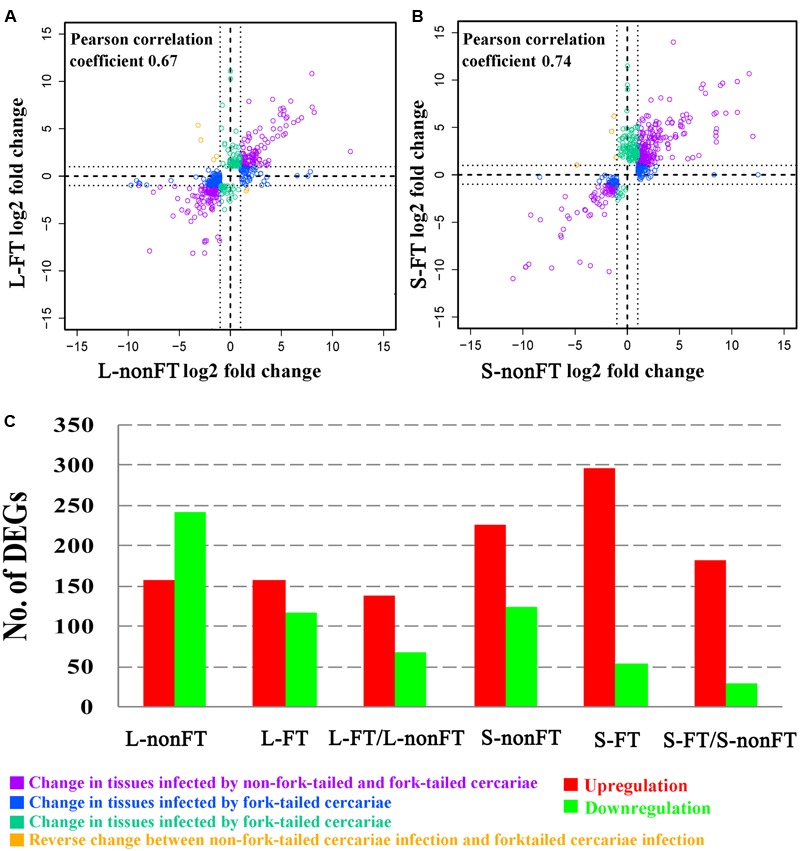
Summary of differentially expressed genes (DEGs). **(A)** Pearson correlation between DEGs of L-nonFT and DEGs of L-FT. **(B)** Pearson correlation between DEGs of S-nonFT and DEGs of S-FT. **(C)** DEGs number in each group. L-nonFT represents liver infected by non-fork-tailed *S. japonicum* cercaria. L-FT represents liver infected by fork-tailed *S. japonicum* cercaria. S-nonFT represents skin infected by non-fork-tailed *S. japonicum* cercaria. S-FT represents skin infected by fork-tailed *S. japonicum* cercaria.

**FIGURE 2:**
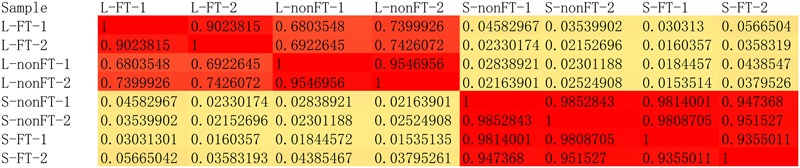
The global pearson correlation coefficient between expressional changes of the two replicates.

According to Agilent2100 Bioanalyzer analysis, the RINs of all RNA samples were greater than 7.0. In each group, Q20 percentage over 95%. About 50 million clean reads were obtained in each sample. More than fifteen thousand genes were detected in present study. 158 upregulated genes and 242 downregulated genes were found in non-fork-tailed *S. japonicum* cercaria infecting liver (L-nonFT). However, 157 upregulated genes and 118 downregulated genes were found in fork-tailed *S. japonicum* cercaria infecting liver (L-FT). 138 upregulated genes and 69 downregulated genes differentially expressed between L-FT and L-nonFT (**Figure 1**). In the infected skins, 225 upregulated genes and 124 downregulated genes were found in non-fork-tailed cercaria infecting skin (S-nonFT). 295 upregulated genes and 54 downregulated genes were found in fork-tailed cercaria infecting skin (S-FT). 182 upregulated genes and 29 downregulated genes differentially expressed between S-FT and S-nonFT. The pearson correlation coefficient of DEGs between L-FT and L-nonFT was 0.67, while the pearson correlation coefficient of DEGs between S-FT and S-nonFT was 0.74. The global gene expression pearson correlation coefficient between the two replicates were range from 0.90 to 0.98 (**Figure 2**). Most Q-PCR results shared the same expressional trends with RNA-seq (**Supplementary Figure S1**).

### GO and KEGG Enrichment Analyses of Infected Livers or Skins

Gene Ontology and KEGG databases were applied for functional annotation and enrichment analysis. In infected livers, 52 GO terms were significantly enriched in both of L-nonFT and L-FT. Most of them are metabolism related GO terms (**Supplementary Data Sheets 1, 2**). Additionally, most enriched metabolism processes were downregulated in L-nonFT group, while upregulated metabolism processes were found in L-FT group. 46 significantly enriched GO terms were found between L-FT and L-nonFT. Most of the 46 GO terms were metabolism related GO terms and the metabolism processes were stronger in L-FT (**Supplementary Data Sheet 3**). Eleven pathways were significantly enriched in L-nonFT, while 21 pathways were significantly enriched in L-FT (**Supplementary Data Sheet 2**). Eighteen significantly enriched pathways were found between L-FT and L-nonFT (**Supplementary Data Sheet 3**) and most of these enriched pathways are involved in metabolism processes. Pathogenic *Escherichia coli* infection pathway was the only significantly enriched pathway which related to infection or immune response in L-nonFT. Caffeine metabolism, sulfur metabolism, porphyrin and chlorophyll metabolism and complement and coagulation cascade were significantly enriched in L-FT only. Toll-like receptor signaling pathway was not significantly enriched in infected liver, however, one key component (Irf7) of toll-like receptor signaling pathway was downregulated in both of L-nonFT and L-FT (**Table 2**).

**Table 2:** Differentially expressed transcription factors in infected livers or skins.

Transcription factor family	Transcription factor	L-nonFT(Log2 fold change)	L-FT(Log2 fold change)	S-nonFT(Log2 fold change)	S-FT(Log2 fold change)
Family:ARID	Arid5b	1.744377	0.255159	-0.10437	-0.22878
Family:bHLH	Arntl	-5.16863	-2.37464	-0.72068	-1.3467
Family:bHLH	Bhlhe41	6.531381	6.228819	1.766867	0.023723
Family:bHLH	Hes6	-0.26462	0.760736	-0.5198	-0.47499
Family:bHLH	Myc	1.401067	-0.51821	0.237499	0.992387
Family:bHLH	Srebf1	-2.0337	-0.69429	1.009808	0.468553
Family:bHLH	Npas2	-5.61738	-4.21883	-0.24101	-3.19143
Family:C/EBP	Cebpb	1.609939	1.018074	-0.12923	0.906537
Family:C/EBP	Cebpa	0.54264	0.504914	0.695452	2.107102
Family:CUT	Onecut1	3.53318	1.118757	No data	No data
Family:ESR-like	Ar	4.459432	6.179909	1.991742	0.387604
Family:ETS	Spi1	-0.75176	-0.80993	1.242256	1.996953
Family:Forkhead	Foxa3	0.459164	-0.59706	-3.45943	-3.45943
Family:Forkhead	Foxq1	0.650976	-1.20901	1.247928	-0.41504
Family:HMG	Sox18	-1.22553	-1.28279	-1.09544	-0.91334
Family:HMGI/HMGY	Hmga1	-0.04477	-2.65896	0.143096	-0.37148
Family:Homeobox	Hopx	1.65774	1.232161	0.184947	-0.60868
Family:Homeobox	Hhex	0.437507	-0.6039	-0.51143	-0.74959
Family:IRF	Irf7	-1.59553	-1.654	-0.11459	3.04564
Family:IRF	Irf1	-0.74215	-0.48373	0.097016	1.704004
Family:IRF	Irf9	-0.99419	-0.99613	-0.24284	1.311586
Family:Miscellaneous	Nr0b2	-0.86414	0.318146	-3.32193	-3.32193
Family:SAND	Sp110	-0.46138	-0.75995	1.119612	2.101329
Family:STAT	Stat1	-0.5378	-0.69545	0.180428	2.79527
Family:STAT	Stat2	-0.72042	-0.67314	0.423419	2.558745
Family:TF-bZIP	Jun	2.362894	0.61728	-0.2881	0.256207
Family:TF-bZIP	Tef	2.225533	2.157897	0.38319	0.872954
Family:TF-bZIP	Nfe2l2	-0.67238	0.35344	0.185609	0.474892
Family:TF-bZIP	Atf5	-1.43063	-0.83637	1.015755	0.475647
Family:TF-bZIP	Maff	2.814493	1.703519	0.589408	2.017427
Family:TF-bZIP	Nfil3	-2.13815	-1.25659	0.182736	0.953936
Family:TF-bZIP	Mafb	2.857215	1.758992	0.837026	1.650973
Family:TF-bZIP	Batf2	-1.52725	-1.97085	0.826548	3.892543
Family:TF-bZIP	Junb	1.562835	0.508301	0.16741	1.051094
Family:TF-bZIP	Fos	1.061164	-0.71256	-1.83582	-2.1009
Family:TF-bZIP	Dbp	6.626142	5.875997	0.838356	2.344737
Family:THR-like	Rorc	-0.59358	-0.1619	1.186474	0.442295
Family:THR-like	Nr1d1	2.450471	2.646729	0.04676	-0.30544
Family:THR-like	Ppard	-3.00331	-2.80956	-0.2797	0.048875
Family:THR-like	Nr1d2	2.293341	2.09211	0.082757	0.45807
Family:TSC22	Tsc22d1	-1.88268	-1.7696	-0.14401	-0.40651
Family:ZBTB	Bcl6	-2.00565	-0.12776	0.105905	0.314873
Family:zf-C2H2	Zfp445	0.710457	1.322984	0.535262	0.274248
Family:zf-C2H2	Gm14420	1.70156	3.722319	0.291651	0.635046
Family:zf-C2H2	Egr1	-0.57262	-3.54102	-0.30641	-1.3635
Family:zf-C2H2	Peg3	-1.91136	-2.84577	0.3109	-0.42065
Family:zf-C2H2	Klf10	0.364352	-0.22098	1.17353	0.182848
Family:zf-C2H2	Klf13	1.4753	1.02966	0.020408	0.329335
Family:zf-C2H2	Gm14308	-0.0977	0.058894	8.301496	6.409391
Family:zf-C2H2	Zkscan7	0.547488	3.083017	0.139474	No data
Family:zf-C2H2	Etohi1	-0.06447	1.513672	0.747907	0.212442
Transcription co-factors	Ifi204	-1.06675	-0.99235	1.208517	2.746205
Transcription co-factors	Ankrd1	No data	No data	5.484695	4.363299
Transcription co-factors	Bcl3	-0.3721	-0.41914	0.950847	2.344383
Transcription co-factors	Calr	-0.96153	-1.2745	0.356958	1.017746
Transcription co-factors	Pnrc1	-1.29687	-0.9161	-0.93507	-0.13759
Transcription co-factors	Ccne1	2.492396	0.892144	0.121991	1.855052
Transcription co-factors	Lpin2	2.163839	2.307111	0.852774	1.169925
Transcription co-factors	Mnda	-1.1882	-1.39102	0.346998	1.760863
Transcription co-factors	Btg1	-1.68806	-0.80647	-0.30055	0.079588
Transcription co-factors	Cry1	-2.1775	-1.80425	0.364456	0.037475
Transcription co-factors	Lmcd1	-1.03171	-1.16601	1.201395	0.36066
Transcription co-factors	Tob1	1.20179	1.301431	-0.14437	-0.23932
Transcription co-factors	Txnip	-0.0122	-0.62252	0.687301	-0.4335
Chromatin remodeling factors	Hdac5	-1.20928	-0.53497	-0.13199	0.09322

In the infected skins, 51 GO terms and 96 GO terms were significantly enriched in S-nonFT and S-FT, respectively (**Supplementary Data Sheet 1**). Sixty-three significantly enriched GO terms were found between S-FT and S-nonFT (**Supplementary Data Sheet 4**). Twenty pathways were significantly enriched in S-nonFT while 36 pathways were significantly enriched in S-FT (**Supplementary Data Sheet 2**). Twenty-six pathways were significantly enriched between S-FT and S-nonFT (**Supplementary Data Sheet 4**). Six pathways (including asthma, phagosome, staphylococcus aureus infection, natural killer cell mediated cytotoxicity, osteoclast differentiation, cell adhesion molecules) were significantly enriched in the three groups (S-nonFT, S-FT and S-FT/S-nonFT).

### Comparative Toxicogenomic Analysis

**FIGURE 3:**
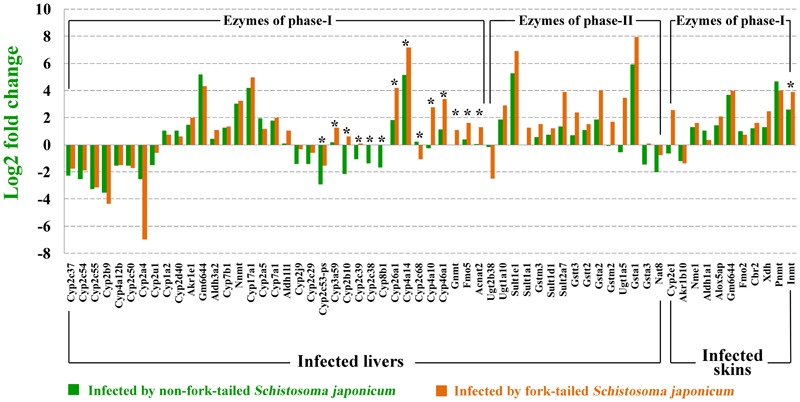
Differentially expressed metabolic enzymes in infected livers or skins. ^∗^Represents differentially expressed genes between non-fork-tailed cercaria infecting tissue and fork-tailed cercaria infecting tissue.

As shown in **Figure 3**, 61 differentially expressed enzymes that involved in detoxification of drugs or xenobiotics were found in infected livers or skins, including 46 enzymes of metabolic pahse I and 15 enzymes of metabolic phase II. Thirteen enzymes significantly expressed higher in the L-FT group comparing with L-nonFT group. Only 1 enzyme (Inmt) expression was significantly higher in S-FT comparing with S-nonFT. No differentially expressed enzyme of metabolic phase II was found in the *S. japonicum* infecting skins. According to Comparative Toxicogenomics Database, thousands drugs or xenobiotics metabolisms will be altered by the differentially expressed enzymes that found in present study, including metabolisms of anti-*S. japonicum* drugs (such as artemisinin and oxadiazoles). Details of relationship between metabolism enzymes and substrate are listed in **Supplementary Data Sheet 5**.

### Cytokine Analysis

**FIGURE 4:**
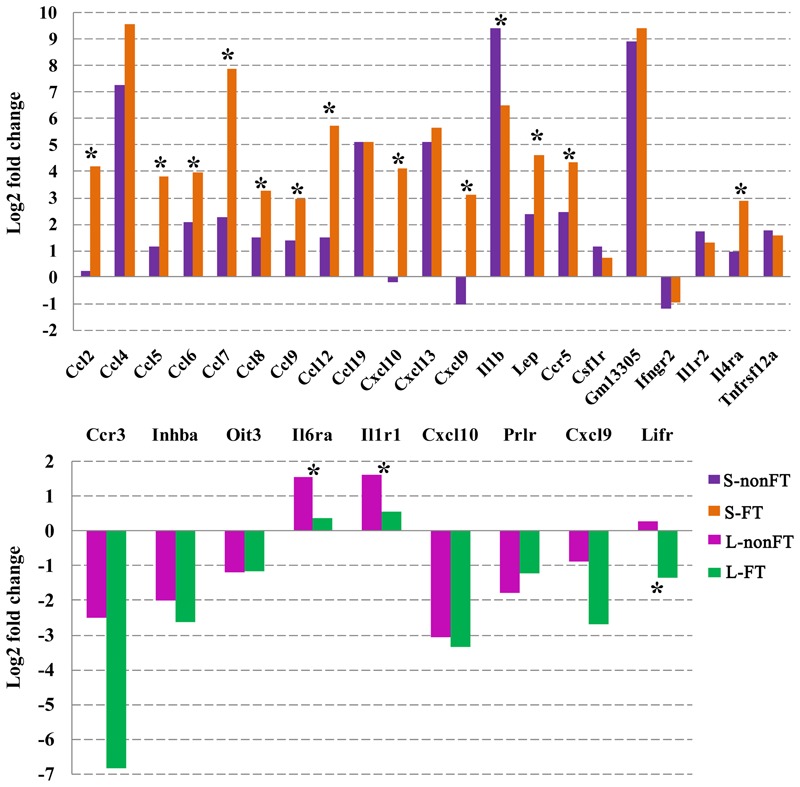
Differentially expressed cytokines and cytokine receptors in infected livers or skins. L-nonFT represents liver infected by non-fork-tailed *S. japonicum* cercaria. L-FT represents liver infected by fork-tailed *S. japonicum* cercaria. S-nonFT represents skin infected by non-fork-tailed *S. japonicum* cercaria. S-FT represents skin infected by fork-tailed *S. japonicum* cercaria. ^∗^Represents differentially expressed genes between non-fork-tailed cercaria infecting tissue and fork-tailed cercaria infecting tissue.

As shown in **Figure 4**, 31 cytokines or cytokine receptors were differentially expressed in infected livers or skins. Ccr3, Oit3, Pxdn, Prlr, Ifngr2, and Inhba were downregulated in infected livers or skins, especially in the infected livers. Cxcl10 and Cxcl9 were downregulated in L-nonFT and S-nonFT, however, upregulations of Cxcl10 and Cxcl9 were found in the S-FT. Il6ra, Il1r1, Il1r2, Ccl8, and Gm13305 were shown upregulated trends in present study. The significant upregulations of Ccl19, Tnfrsf12a, Cxcl13, Ccl12, Ccl4, Lep, Ccl9, Il1b, Ccl6, Csf1r, Ccl7, Ccr5, Ccl5, Ccl2, and Il4ra were only found in the infected skins. Expressions of Ccl2, Ccl5, Ccl6, Ccl7, Ccl8, Ccl9, Ccl12, Cxcl10, Cxcl9, Lep, Ccr5, and Il4ra were significantly higher expressed in S-FT comparing with S-nonFT. Il6ra and Il1r1 were significantly higher expressed in L-nonFT comparing with L-FT.

### Transcription Factor Analysis

**FIGURE 5:**
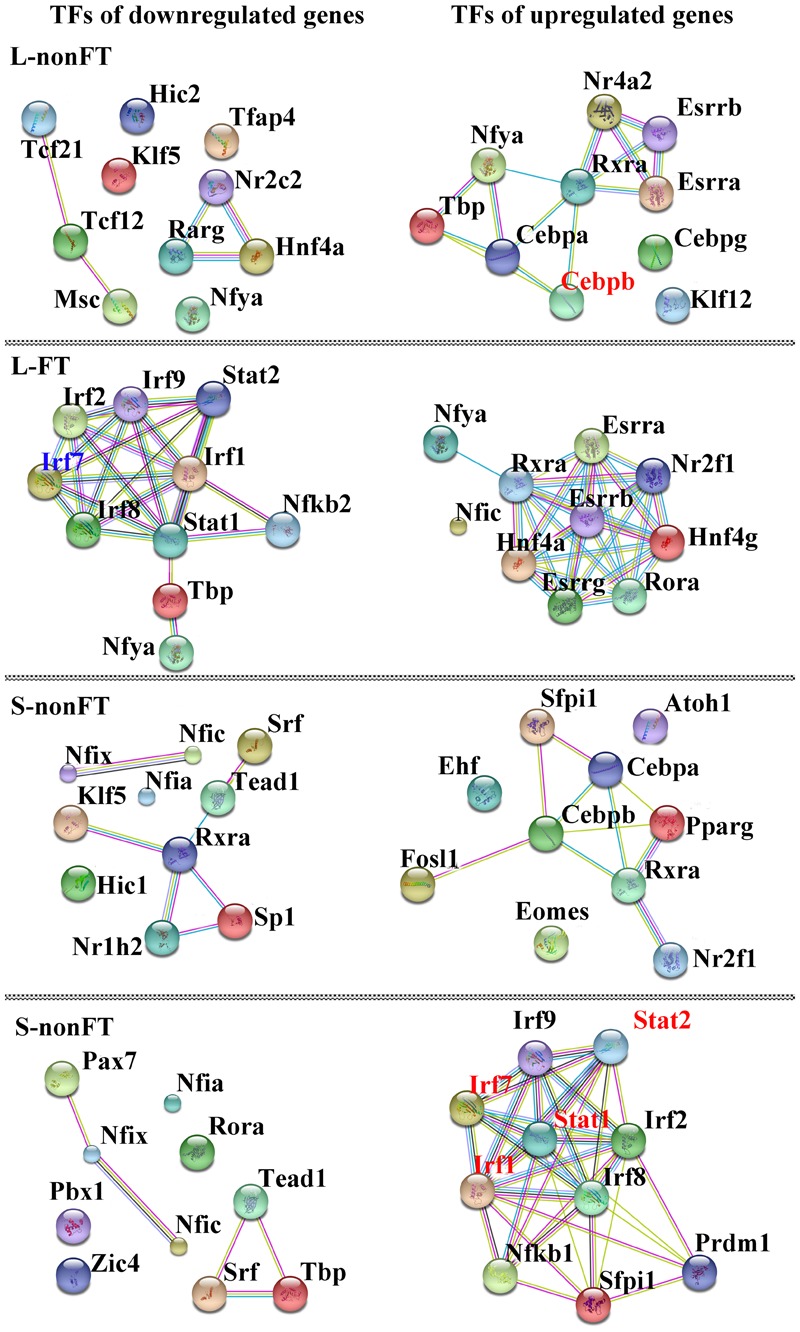
Interactions between top10 TFs used by upregulated genes or doweregulated genes. L-nonFT represents liver infected by non-fork-tailed *S. japonicum* cercaria. L-FT represents liver infected by fork-tailed *S. japonicum* cercaria. S-nonFT represents skin infected by non-fork-tailed *S. japonicum* cercaria. S-FT represents skin infected by fork-tailed *S. japonicum* cercaria. Red presents upregulated gene.

**FIGURE 6:**
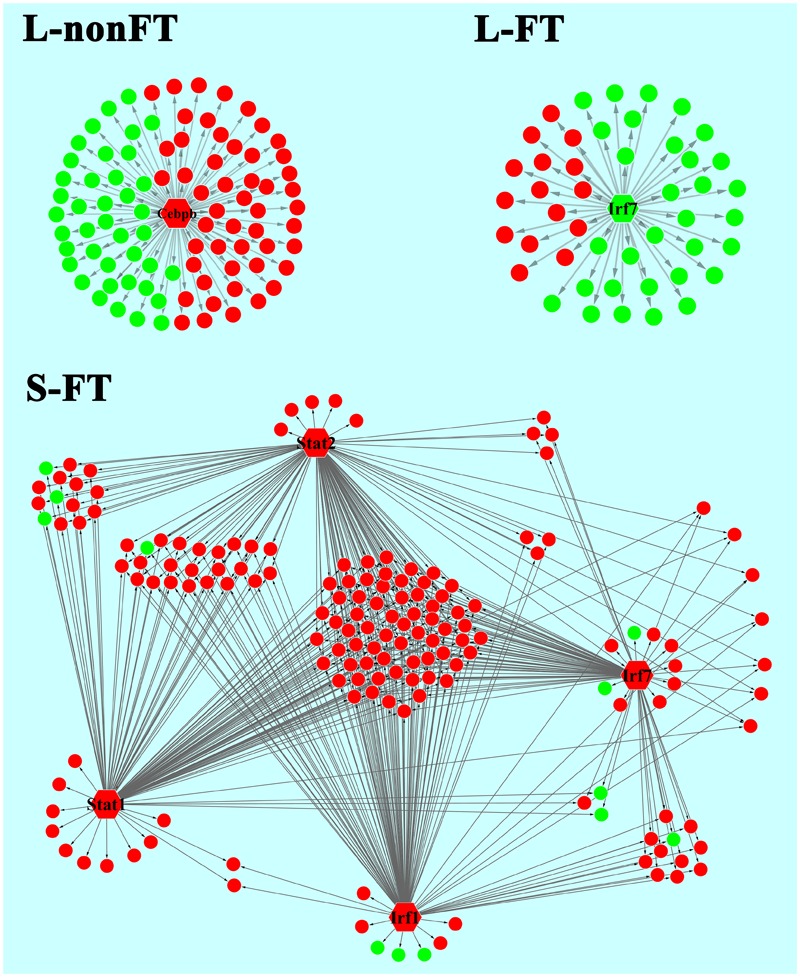
Relationship between differentially expressed TFs and the regulating genes. L-nonFT represents liver infected by non-fork-tailed *S. japonicum* cercaria. L-FT represents liver infected by fork-tailed *S. japonicum* cercaria. S-FT represents skin infected by fork-tailed *S. japonicum* cercaria. TF is presented as hexagon and pie presents the TF regulating gene that differentially expressed in present study. Red presents upregulated gene and green presents downregulated gene.

In present study, as shown in **Table 2**, 65 differentially expressed transcription factors were found in infected livers or skins. These differentially expressed transcription factors can be clustered into 21 transcription factor families, including ARID, bHLH, C/EBP, CUT, ESR-like, ETS, Forkhead, HMG, HMGI/HMGY, Homeobox, IRF, Miscellaneous, SAND, STAT, TF-bZIP, THR-like, TSC22, ZBTB, zf-C2H2, transcription co-factors and chromatin remodeling factors. According to Z-score of Pscan analysis, top 10 transcription factors used by upregulated genes or downregulated genes of each group are shown in **Figure 5**. Some of these transcription factors were differentially expressed in present study, including five upregulated transcription factors (Cebpb of L-nonFT group, Stat1, Stat2, Irf1, and Irf7 of S-FT group) and 1 downregulated transcription factor (Ifr7 of L-FT group). **Figure 6** shows the differentially expressed target genes of the differentially expressed transcription factors.

## Discussion

In present study, all RNA integrity numbers (RIN) were >7.0. Previous report showed that RIN >7.0 is the precondition that ensures the quality of RNA-seq (Romero et al., 2014). Q20 percentage over 95% indicated that our sequencing quality was good enough for ensuring the transcriptomic analysis. The pearson correlation coefficient between the replicates were range from 0.90 to 0.98 (**Figure 2**). These analyses suggested that variability between the replicates was relatively low and the sequencing data was reliable. As shown in **Figure 1C**, most of the differentially expressed genes were upregulated in L-FT, S-nonFT, and S-FT, excepting the mouse liver infected by non-fork-tailed *S. japonicum* cercaria (L-nonFT). Comparison analysis between fork-tailed cercaria infection and non-fork-tailed cercaria infection showed that 138 upregulated genes and 69 downregulated genes were found between L-FT and L-nonFT, 182 upregulated genes and 29 downregulated genes were found between the S-FT and S-nonFT. The correlation between differentially expressed genes of L-nonFT and differentially expressed genes of L-FT was 0.67, while the correlation between differentially expressed genes of S-nonFT and differentially expressed genes of S-FT was 0.74 (**Figures 1A,B**). These results showed that morphology distinct cercariae of *S. japonicum* can induce some different host responses.

Several scientists have reported that *S. japonicum* infection result in metabolic disturbance in the infected liver (Olds et al., 1986; Chen et al., 2002; Wang et al., 2006), however, no comparative analysis has been performed to analyze the differences between fork-tailed *S. japonicum* cercaria infecting tissues and non-fork-tailed *S. japonicum* cercaria infecting tissues. According to GO enrichment analyses, most significantly enriched GO terms of L-nonFT or L-FT were metabolism related, such as lipid metabolic process, small molecule metabolic process, secondary metabolic process and arachidonic acid metabolism. The details of GO enrichment analysis are listed in **Supplementary Data Sheet 1** which shows that most DEGs of each GO term of L-nonFT were downregulated, however, most DEGs of each GO term of L-FT were upregulated. The results of KEGG enrichment analysis consistent with GO enrichment analysis (**Supplementary Data Sheet 2**). These suggested that both of the two types *S. japonicum* cercariae induced hepatic metabolic changes, however, the final results of infections were different. Metabolic processes of non-fork-tailed cercaria infecting liver will be downregulated while metabolic processes of fork-tailed cercaria infecting liver will be upregulated.

Liver is a central organ that in charge of the detoxification of drugs or xenobiotics in mammal. Generally, the detoxification process in liver occurs in three alexipharmic phases including phase-I, phase-II, and phase-III. Phase-I is mainly mediated by cytochrome P450s, aldo-keto reductases, aldehyde dehydrogenases, flavin monooxygenases and other metabolic enzymes. After the drugs or xenobiotics are degraded by metabolic enzymes of phase-I, the products of phase-I will be further processed by enzymes of phase-II, such as glutathione *S*-transferases, UDP-glucuronosyltransferases, sulfotransferases, methyl transferases, and other enzymes. In the phase-III, all detoxified products of phase-II will be exported to intestinal tract through bile secretion and then excreted by excretory organs. In present study, 61 differentially expressed genes that involved in detoxification of drugs or xenobiotics were found in infected livers or skins. As shown in **Figure 3**, fork-tailed *S. japonicum* cercaria infection generally induced higher expressions of detoxifying enzymes, especially the enzymes of phase-I. According to Comparative Toxicogenomics Database, some medicines used for anti-*S. japonicum* were also targeted by the differentially expressed enzymes that were found in present study. For example, artemisinin and oxadiazoles can be used for treatment of *S. japonicum* infection (Fenwick and Webster, 2006; Sayed et al., 2008). In present study, Cyp2a5, which in charges of artemisinin metabolism, was upregulated in infected livers. The expressions of Gsta3 and Gsta1 that involved in oxadiazoles metabolism were also showed altered expressions in infected livers. The present results suggested that *S. japonicum* infection can alter host pharmacokinetics and then influences the effects of medical care. We don’t know whether there are substances listed in **Supplementary Data Sheet 5** will contribute to anti-*S. japonicum.* As the developing of the medicinal development, more and more new anti-*S. japonicum* drugs will be found, our comparative toxicogenomics analysis results (**Supplementary Data Sheet 5**) could provide valuable data for studying pharmacokinetics of rising anti-*S. japonicum* drugs and then contributes to treatment of *schistosomiasis*.

In contrast with infected livers, most significantly enriched GO terms or KEGG pathways of infected skins were immunity related, such as response to cytokine stimulus, antigen processing and presentation, extracellular region and MHC protein complex (**Supplementary Data Sheets 1, 2**). MHC complex plays an essential role in innate immune response against *S. japonicum* (Waine et al., 1998; Tang et al., 2012; Chen et al., 2017a). In present study, MHC complex was significantly upregulated in the infected skins, however, MHC complex related GO terms was not significantly enriched in infected livers. Additionally, as shown in **Supplementary Data Sheets 1, 2**, only a few immunity related GO terms were enriched in infected livers. So *S. japonicum* cercaria infection showed different impacts on host liver and skin. *S. japonicum* cercaria infection in the liver influences host metabolism mainly and non-fork-tailed cercaria infection showed much lower metabolic ability comparing with fork-tailed cercaria infection, however, the *S. japonicum* cercaria infection in the skin influences host immune response mainly and the host skin showed much stronger immune response during fork-tailed *S. japonicum* cercaria infection (**Figure 4**).

Host immune response is connected with cytokine pathway closely. So analysis of cytokine pathway change during *S. japonicum* infection is necessary for us to understand the battle between host and *S. japonicum*. As shown in **Figure 4**, only nine cytokines or cytokine receptors were differentially expressed in infected livers. The cytokine expressional changes of L-nonFT and L-FT were similar, however, Lifr was downregulated in L-FT. Lifr is involved in suppression of granuloma formation (Okamura et al., 2010). The downregulation of Lifr in L-FT suggested that granuloma could be much more serious in the liver during for-tailed cercaria infection. Cxcl10 and Cxcl9 were downregulated in both L-nonFT, L-FT, and S-nonFT. According to published report, Cxcl9 and Cxcl10 are involved in recruitments of neutrophil, Th1, B cell, dendritic cell and eosinophil (Griffith et al., 2014). It suggested that chemotaxis of neutrophil, Th1, B cell, dendritic cell and eosinophil will be downregulated in the infected livers, regardless of biotype of *S. japonicum* cercaria. Although most differentially expressed cytokines or cytokine receptors were downregulated in infected livers, the infected skins showed an enhanced inflammation response. In present study, 21 cytokines or cytokine receptors were differentially expressed in infected skins. Most of the differentially expressed cytokines or cytokine receptors of infected skins were upregulated during *S. japonicum* infection, including Ccl2, Ccl4, Ccl5, Ccl6, Ccl7, Ccl8, Ccl9, Ccl12, Ccl19, Cxcl13, Il1b, Ccr5, Gm13305, Il1r2, and Tnfrsf12a. Additionally, most of these cytokines or receptors showed higher expressions in S-FT comparing with S-nonFT (**Figure 4**). According to published report (Griffith et al., 2014), Ccl19, Cxcl13, Ccl12, Ccl4, Ccl9, Ccl6, Ccl7, and Ccl2 involved in recruitments of T cell, Th1, Th2, B cell, DC, immature DC, NK, astrocyte, basophil, eosinophil, monocyte, macrophage, mast cell and neutrophil. So the present RNA-seq results indicated that the inflammatory responses in the infected skins were stronger than that of infected livers, especially in S-FT group. Published reports showed that IL-4 and IL-13 were upregulated during *S. japonicum* infection (Oswald et al., 2001; Bartley et al., 2006), however, both of these cytokine showed no differential expression in present study. The inconsistent could be the results of different host, sample collection time, detection technologies, criterion of differentially expressed gene or differential infection progresses. Although IL-4 showed no differentially expressed in present study, its receptor Il4ra was upregulated in the infected mouse skins, especially in the fork-tailed *S. japonicum* cercaria infecting skin (**Figure 4**). It is clear that Il4ra is one of the key components of IL-4 signaling pathway which involved in recruitment of mediators of cell growth, cellular resistance to apoptosis, gene activation and immune cell differentiation (Nelms et al., 1999). So, in present study, it is very likely that upregulation of Il4ra will enhance the effects of IL-4 signaling pathway under the condition that IL-4 shows no differentially expressed.

Expression of cytokine is regulated by various transcription factors, such as signal transducer and activator of transcription (Stat) and interferon regulatory factor (Irf). These transcription factors play important roles in host inflammation induction (Taniguchi et al., 2001; Hanada and Yoshimura, 2002). STAT1-/- mice shows decreased cytotoxicity of NK cell (Chen et al., 2016) which negatively regulates *S. japonicum* egg-induced liver fibrosis (Hou et al., 2012). Stat2 is one of the TFs in STAT signaling pathway which regulates host immune response (Decker et al., 2002), however, its role in *S. japonicum* infection still unclear. Irf7 has been confirmed as important regulator of innate immune response (Lu et al., 2002), however, its role in *S. japonicum* infection is needed to be illustrated too. In present study, 65 differentially expressed TF were found in infected livers or skins, including Stat1, Stat2, and Irf7 (**Table 2**). These 65 differentially expressed TFs can be clustered into 21 transcription factor families. As shown in **Table 2**, the members of transcription co-factors family and TF-bZIP family were affected most by *S. japonicum* infection and there were 13 transcription co-factors and 11 TF-bZIPs differentially expressed.

Pscan was applied to analyze TF regulating genes and String database was applied to analyze the interactions between the TFs. According to *Z*-score of Pscan analysis, top 10 TFs used by upregulated genes or downregulated genes are shown in **Figure 6**. As shown in **Figure 6**, there were five differentially expressed TF, including Cebpb, Stat1, Stat2, Irf1, and Irf7 (**Figure 6**). Cebpb, which was upregulated in present study, is a key hepatocyte TF which is known to regulate the function or regeneration of animal liver (Jakobsen et al., 2013). Irf7 is involved in regulation of type I interferon production, oncogenesis and apoptosis (Ning et al., 2011). In present study, Irf7 was downregulated in L-FT group. The downregulation of Irf7 consistent with that most of the DEGS that are regulated by Irf7 were downregulated in L-FT group (**Figure 6**). Irf7 was upregulated in S-FT group, it also agreed with that most DEGs that are regulated by Irf7 were upregulated in fork-tailed cercaria infecting skin (**Figure 6**). So, during the fork-tailed *S. japonicum* cercaria infection, it is very likely that the differential expression of Irf7 plays an important regulating role in host gene expressional regulation and then contributes to host immune responses against *S. japonicum* infection. Beside of Irf7, three upregulated TFs (Stat1, Stat2, and Irf1) were found in S-FT group. It also consistents with that most DEGs that are regulated by Stat1, Stat2, and Irf1 were upregulated in S-FT group, especially those genes regulated by Stat1, Stat2, Irf1, and Irf7 at the same time. These results indicated that *S. japonicum* can alter host TF expression and then regulates host biological processes indirectly. The differentially expressed TFs, such as Stat1, Stat2, Irf1, and Irf7, could play important regulatory roles during *S. japonicum* infection.

## Conclusion

In present study, for the first time, we analyzed the transcriptomic changes of mouse livers and skins infected by non-fork-tailed *S. japonicum* cercaria or fork-tailed *S. japonicum* cercaria, analyzed the transcriptomic differences between the two cercariae infecting tissues. The present results will give us valuable data about the gene expressional differences between non-fork-tailed *S. japonicum* cercaria infecting tissues and fork-tailed *S. japonicum* cercaria infecting tissues. Our results indicate that, during the *S. japonicum* infection, host liver and skin show totally different transcriptomic landscapes. The functional changes of infected livers focus on alterations of metabolic processes. Additionally, non-fork-tailed *S. japonicum* cercaria infecting liver shows lower metabolic ability comparing with fork-tailed *S. japonicum* cercaria infecting liver. The gene expressional changes of infected skins focus on upregulation of inflammation response. Furthermore, fork-tailed cercaria infecting skin shows much stronger inflammation response than that of non-fork-tailed cercariae infecting skin. Comparative Toxicogenomic analysis shows that *S. japonicum* infection can alter the expressions of genes that involved in regulating pharmacokinetics of host (including pharmacokinetics of some anti-*S. japonicum* drugs). Transcription factor analysis reveals that some TF expressions can be altered during *S. japonicum* infection and the TF usage frequency depend on biotypes of *S. japonicum* cercariae and infected tissue attributes. Irf7, Stat1, and Stat2 could play important regulation roles in host inflammatory response during fork-tailed *S. japonicum* cercaria infection.

## Author Contributions

F-CZ and J-JH conceived and designed the study, and critically revised the manuscript. YY, SH, and XX performed the experiments, analyzed the data and drafted the manuscript, J-FY and HC helped in study design, study implementation and manuscript revision. All authors read and approved the final manuscript.

## Conflict of Interest Statement

The authors declare that the research was conducted in the absence of any commercial or financial relationships that could be construed as a potential conflict of interest.

## Footnotes

1 http://ctdbase.org/

## Supplementary Material

FIGURE S1 Quantitative PCR (Q-PCR) validation of the results of RNA-seq.

DATA SHEET 1 Significantly enriched GO terms.

DATA SHEET 2 Significantly enriched pathways.

DATA SHEET 3 Results of DEG comparative enrichment analysis between L-nonFT and L-FT.

DATA SHEET 4 Results of DEG comparative enrichment analysis between S-nonFT and S-FT.

DATA SHEET 5 Relationship between differentially expressed metabolism enzyme and substrate.
