# Supplementary figures and images for: Comparative Study of Transcriptome Profiles of Mouse Livers and Skins Infected by Fork-Tailed or Non-Fork-Tailed Schistosoma japonicum
Source: Front Microbiol. 2017 Aug 30;8:1648. doi: 10.3389/fmicb.2017.01648 (PMC5582157; doi:10.3389/fmicb.2017.01648)

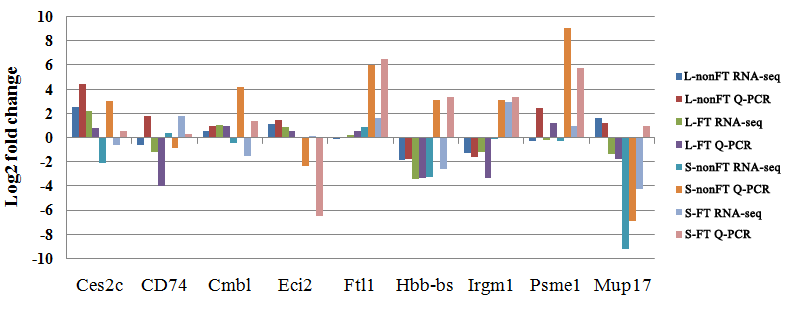

Supplement: FIGURE S1 — Quantitative PCR (Q-PCR) validation of the results of RNA-seq. [file Image_1.tif]
